# Identifying Indicators of Smartphone Addiction Through User-App Interaction

**DOI:** 10.1016/j.chb.2019.04.023

**Published:** 2019-10

**Authors:** Beryl Noë, Liam D. Turner, David E.J. Linden, Stuart M. Allen, Bjorn Winkens, Roger M. Whitaker

**Affiliations:** a School of Computer Science and Informatics, Cardiff University, 5 The Parade, CF24 3AA, Cardiff, UK; b School for Mental Health and Neuroscience, Faculty of Health, Medicine and Life Sciences, Maastricht University, Universiteitssingel 40, 6229 ET Maastricht, the Netherlands; c Department of Methodology & Statistics, Care and Public Health Research Institute (CAPHRI), Faculty of Health, Medicine and Life Sciences, Maastricht University, Universiteitssingel 40, 6229 ET Maastricht, the Netherlands

**Keywords:** Smartphone addiction, Smartphone usage, User interface, Device interaction, Snapchat, Social media

## Abstract

We introduce a new approach to monitoring the activity of smartphone users based on their physical interactions with the interface. Typical events are taps, scrolling and typing, carried out to interact with apps. As compared to other measures, this directly encapsulates potential problematic physical smartphone behaviour as a signal. The approach contrasts against conventions such as self-reporting or timing activity sessions, and it focusses on active rather than passive smartphone activity. Using this alternative method, we collected all user interface interaction events from a sample of 64 participants over a period of 8 weeks, using a bespoke monitoring app called Tymer. User Smartphone Addiction was seen to significantly correlate with high levels of interaction with Lifestyle apps, particularly for female users. Interactions with Social apps in general were also associated with Smartphone Addiction. In particular, user interactions with Snapchat correlated with Smartphone Addiction, represented across all types of interface interaction. This is significant given the widespread usage of Snapchat by teenagers, and we hypothesise that the app's design provides a particularly strong pathway in support of Smartphone Addiction.

## Highlights

• We assess the link between users' device interactions and Smartphone Addiction.
• The approach distinguishes between passive and active Smartphone usage.
• Lifestyle apps are associated with Smartphone Addiction, especially for female users.
• Social apps in general are associated with Smartphone Addiction.
• User interactions with Snapchat strongly correlate with Smartphone Addiction.

## 1 Introduction

Smartphone usage is now ubiquitous across much of the global population, with the smartphone offering a vast range of applications (apps) that help to extend human cognition (Clark & Chalmers, 1998). Typical daily usage levels are high (De-Sola Gutiérrez et al., 2016) and there is evidence of dependency and attachment to smartphone technology (Hoffner, Lee, & Park, 2016), combined with the potential disruptiveness of incoming notifications (Turner et al., 2015, 2017) and “checking habit” formation (Oulasvirta, Rattenbury, Ma, & Raita, 2012). It is now acknowledged that such uncontrolled and problematic behaviour can become harmful, being recognised as *Smartphone Addiction* (SA) (Pearson & Hussain, 2016). Problematic smartphone use has been linked to teenage depression and anxiety (Ha, Chin, Park, Ryu, & Yu, 2008; Lemola et al., 2014), and more widely, various relationships have been found concerning stress, depression, sleeping problems, anxiety, subjective well-being, and loneliness (Demirci, Akgönül, & Akpinar, 2015; Elhai, Dvorak, Levine, & Hall, 2017; Lee, Chang, You, & Cheng, 2014; Lemola et al., 2014).

Despite this research progress, detecting indicators of problematic smartphone behaviour is a challenge for two main reasons. Firstly, the range of utility that smartphone apps provide means that usage levels are generally high and that high usage is socially acceptable (Chotpitayasunondh and Douglas, 2016). Therefore behaviour correlating with SA can easily be hidden. Secondly, apps increasingly allow the smartphone to be passively used for large periods of time as a substitute for other devices (e.g., GPS navigation, TV, music player) meaning that as smartphone usage gets more diverse, high level metrics, such as time on the smartphone, may not represent the strongest indicator of problematic behaviour. These issues contribute to the invisibility of SA (Roberts et al., 2014) and the challenge of encouraging behaviour change to avoid it.

In this paper, we propose a new approach to identifying behaviours indicative of SA. By examining *all* interactions that a user performs through physically touching the screen (e.g., typing, scrolling, tapping), we are able to consider detailed user-app interactions as an indicator for SA. This allows us to gain new insight into the potential sources of problematic behaviour and pathways to addiction. In particular, our approach is based on measures of *active* usage than *passive* usage, which is determined by the extent of user engagement with the interface while the smartphone is operational. It also avoids the need for self-reporting and goes beyond the convention of monitoring screen or app time. Validating the approach required the development of a bespoke smartphone application called *Tymer*, which listens for and records the smartphone events that are triggered by human interaction, which was deployed on a cohort of 76 recruited participants over an eight week period (Noë, Turner, Linden, Allen, Maio, & Whitaker, 2017). Unlike previous work, this approach allows us to examine a wide range of apps within a single study, and make relative comparisons between different apps and classes of apps.

## 2 Related literature and hypotheses

Smartphone Addiction is an extension of Internet Addiction (Kimberly, 1998), which may also encompass other forms of behaviour bundled through the device, such as gaming, social networking, and online shopping (Block, 2008; Montag et al., 2015a). SA broadly refers to the condition leading to uncontrolled smartphone use despite the experience of negative repercussions on personal and social life. From an initial focus on the “cell-phone” as a means to communicate (e.g. (James & Drennan, 2005; Park, 2005)), the functionality provided by the latest generation of smartphones allows untethered Internet access, with applications optimised for mobile usage. The first smartphones with large touch-screens became globally available little over a decade ago: Apple's *iPhone* in 2007, followed by HTC's *Dream* in 2008, with the first studies on SA being published in 2011 (Chae and Lee, 2011; Woojae, Park, & Chang Lee, 2011).

Since then, interest in SA has grown strongly. Although, it has not yet received the clinical acknowledgement from the World Health Organisation (WHO) or American Psychiatric Association (APA), the WHO recently added gaming disorder to the ICD-11 (World Health Organization, 2018). Behavioural addictions share many similarities to substance abuse disorders, but differ inherently in the way they are caused not by a substance, but by a specific behaviour that is experienced as rewarding despite any associated negative consequences (Alavi et al., 2012). As such, it has been argued that any stimulating experience can potentially become addictive (Stanton & Brodsky, 1975).

Assessments for SA have resulted in various self-diagnostics (Kwon et al., 2013, Kwon, Kim, Cho, & Yang, 2013; Lin et al., 2014; Tossel, Kortum, Shepard, Rahmati, & Zhong, 2015), with the Smartphone Addiction Scale in particular combining assessment of substance use disorders, Internet Addiction, and the smartphone's own features. Current research related to SA frequently considers a particular application (e.g., Facebook (Ryan, Chester, Reece, & Xenos, 2014), WhatsApp (Montag et al., 2015b)) or a class of apps (e.g., social networking (Salehan & Negahban, 2013)). In this context it is important to note that SA can, like Internet Addiction (Griffiths, 2012), be argued to be both source and pathway to the causes of addiction.

Measures of smartphone activity typically involve self-reporting (e.g. (Elhai, Levine, Dvorak, & Hall, 2016)) or recording screen time (e.g. (Ferreira, Goncalves, Kostakos, Barkhuus, & Dey, 2014)). Although valid and worthy, these approaches have limitations, such as allowing subjects to filter their own reports of behaviour (King & Bruner, 2000) or including passive usage. Additionally, important details can be obfuscated that a user may consider insignificant, such as a dependency on a particular type of physical interaction with the device, or excessive repetition. Automated collection of user-data sets a new standard (Stachl et al., 2017), but this is more challenging to achieve and less well-established.

These issues motivate the new approach taken in this paper, which goes beyond monitoring high level metrics such as time on the device, to understand how the detailed interactions with the smartphone may coincide with SA. Furthermore, different apps, and different types of smartphone interaction may present different potential pathways to SA and different levels of risk. Our approach involves automatically recording all of the user's interactions with the user interface (UI) for all their applications and activity, over an eight week period. Related work taking this approach is scant: although tracking device interaction is an established technique in the human-computer interaction literature (Cao & Lin, 2017; Jesdabodi & Maalej, 2015; Peng, Zhao, & Zhu, 2016; Shin & Dey, 2013), detailed assessment of correlations with SA are limited, with usage typically being assessed at the higher level (e.g., time on device (Falaki et al., 2010; HintzeRainhard et al., 2014)) and correlated with other aspects such as personality (Gokul, Blom, & Gatica-Perez, 2013; Stachl et al., 2017) and mood (LiKamWa, Liu, Lane, & Zhong, 2013).

### 2.1 Motivating issues

Historically, high usage, measured by time, has been shown to be a key element of SA (e.g. (Bae, 2017; De-Sola Gutiérrez et al., 2016; Lee et al., 2014a; Lemola et al., 2014; Roberts et al., 2014; Rozgonjuk, Levine, Hall, & Elhai, 2018)). Differences in time spent on smartphones is often attributed to habit (Oulasvirta et al., 2012; Tossel et al., 2015) and usage frequency (Tossel et al., 2015). However, as smartphone capabilities continue to increase and support a wider variety of apps, it becomes important to determine the extent to which SA is related to particular types of use. Active usage, where the user is engaged through UI interactions, as compared to passive use (e.g., video streaming), remains an important distinction. To the best of our knowledge, this distinction has not been studied in the literature, due to the additional complexity required in assessing a user's interactions at a granular level. We focus on this and determine the interaction events occurring at the user interface. Based on previous observations regarding time, it is appropriate to hypothesise that *high-scorers in SA have more UI interactions than low-scorers* (H1); this can be considered across all applications.

It is challenging to assert with confidence how SA may manifest itself in terms of specific types of UI interactions, relative to the broader user population. Social media has been reported as one of the top reasons for smartphone use. In particular, social networking applications were found to be the second most downloaded type of application after games (Lim et al., 2015) and social networking accounted for the most application launches, representing 18% of the total number of launches in front of browser and search launches at 14% in recent assessments (Carrascal & Church, 2015). According to Statista (Statista, 2018), daily time spent on social networking is on the rise, reaching an estimated 135 min in 2017, representing an eighth of the time spent awake by an average adult. Social media has also been linked to SA in teenagers (Jeong, Kim, Yum, & Hwang, 2016) and adults (Enez Darcin et al., 2016). Large networks and high participation intensity in networks seem to contribute to the use of social media apps and SA (Salehan & Negahban, 2013). Facebook (Beyens, Frison, & Eggermont, 2016; Roberts et al., 2014; Ryan et al., 2014), Twitter (Roberts et al., 2014), and Instagram (Roberts et al., 2014) in particular have been identified as social media platforms whose use seems associated with SA. Recent work (Punyanunt-Carter et al., 2017), examining the user's relationship with the Snapchat social media app has posited that a user's functional and entertainment needs correlate with Internet Addiction. Interestingly, social media addiction also had the highest correlation to Internet Addiction (Montag et al., 2015a) in comparison to a number of addictions to other online activities: gaming, shopping, social networks, and pornography. *We therefore hypothesise that high-scorers in SA have more UI interactions on social media applications* (H2), with particular reference to Facebook, Instagram and Snapchat (Beyens et al., 2016; Punyanunt-Carter et al., 2017; Roberts et al., 2014; Ryan et al., 2014). This translates as a greater number of UI interactions registered across the “Social” apps category.

Closely related to social media, the communication function of smartphones has been retained as one of the central activities for users (Böhmer, Hecht, Johannes, Krüger, & Bauer, 2011; Carrascal & Church, 2015), despite the extended role of the smartphone as a pocket computer. Messaging (Lee et al., 2014a; Lemola et al., 2014; Yen et al., 2009) and phone calls (Roberts et al., 2014; Yen et al., 2009) have previously been associated with SA. Texting has also been found to be positively related to the mobile phone interfering with life (David, Kim, Brickman, Ran, & Curtis, 2015). In comparison to Facebook however, little evidence for links to SA have been found for Facebook Messenger and WhatsApp, two of the most popular communication apps worldwide. Overall, the messaging literature points to supporting the proposition that *high-scorers in SA will have more UI interactions* supporting *communication activity* (H3), detectable in our context from a larger number of user-generated events on the “Communication” apps category.

### 2.2 Summary of hypotheses

The hypotheses involve testing the extent to which UI interactions, at the most granular level possible, are indicative of SA. In summary:

H1 SA score is positively associated with the number of UI interactions with the device.

H2 SA score is positively associated with the number of UI interactions on apps of the Social app category, with particular reference to Facebook, Instagram, and Snapchat.

H3 SA score is positively associated with the number of UI interactions on apps of the Communication app category, with particular reference to WhatsApp and Facebook Messenger.

To the best of our knowledge, these hypotheses have not been previously tested and therefore the significance of UI interactions as an indicator of SA has not been established. We address the hypotheses in the context of an exploratory study, where wider observations are also presented. We investigate the hypotheses from three perspectives: the different types of UI interactions, as detected through machine-readable actions; the UI interactions with different apps and the UI interactions with different categories of apps. This also allows us to gain new potential insight into the sources of problematic behaviour and pathways to addiction. The specific interactions generated relate to tapping (short and long), writing, scrolling and text selection.

## 3 Methods

Seventy-six participants were recruited through posters and online advertisement at Cardiff University, UK. Participants were required to own a smartphone running Android 4.4 (KitKat) or higher and to have no history of mental illness. Composition of the full sample is presented in Table 1. Due to the voluntary nature of the participation in this study and the lack of control we had over participants' phones, certain participants were excluded as too little or no usage data was collected from them (e.g., due to the lack of space on their phone to store any data), resulting in the consideration of 64 participants in total. Thirty-four participants were male and 30 female, and they were aged between 19 and 46 (M=25.44,SD=5.87). A large sub-group are students (79%), who together with adolescents frequently make up samples for academic studies in this field (De-Sola Gutiérrez et al., 2016), consistent with their propensity to use smartphones. A significant proportion (41%) were employed.

**Table 1:** Sociodemographic characteristics and Smartphone Addition scores (N = 64).

**Age**	**M**	**SD**
Years	25.4	5.87
**Gender**	**N**	**%**
Male	34	53.13
Female	30	46.88
**Employment**	**N**	**%**
Student	38	59.38
Student & employed	13	20.31
Employed	12	18.75
Unemployed	1	1.56
**Education**	**N**	**%**
High school, no diploma	1	1.56
High school diploma or the equivalent	5	7.81
Trade/technical/vocational training	1	1.56
Some undergraduate education, no degree	14	21.88
Bachelor's degree	19	29.69
Master's degree	21	32.81
Doctorate	2	3.13
No answer	1	1.56
**Smartphone Addiction Scale -long version**	**M**	**SD**
Score	87.8	20.26
**Smartphone Addiction Scale -short version**	**N**	**%**
Addicted	12	18.75
Not addicted	52	81.25

All participants were required to attend a briefing and a debriefing session. In the briefing session, participants gave their informed consent and downloaded our custom smartphone application Tymer. They were introduced to its functionality and were asked to keep the app installed and use it over a period of 8 weeks (56 days). In the debriefing session, participants received monetary compensation. In both briefing and debriefing sessions, participants were asked to complete 5 surveys: the Smartphone Addiction Scale (SAS) (Kwon et al., 2013b), the Positive And Negative Affect Schedule (PANAS) (Watson & Clark, 1994), the Big Five Inventory (BFI) (John & Srivastava, 1999), the Monetary Choice Questionnaire (MCQ) (Kirby & Petry, 2004), and a demographics and smartphone use questionnaire. The Smartphone Addiction Scale was the focus of assessment in this particular work, which is part of a wider set of analyses (Noë et al., 2017; Turner et al., 2019).

The SAS (Kwon et al., 2013b) was given the highest reliability score of the scales evaluated by De-Sola Gutiérrez and colleagues in their review (De-Sola Gutiérrez et al., 2016) and presents 33 questions each using a 6-point Likert scale. Total possible scores therefore range from 33 to 198, with a higher score indicating a higher likelihood of addiction. In our analyses, we use the score as a discrete variable. A shortened version (SAS-SV), composed of a subset of 10-questions is also available (Kwon et al., 2013a), for which the cut-off values of 31 for male and 33 for female participants have been tentatively proposed to determine SA. The responses of our participants to this subset of questions were therefore also evaluated separately and 18.75% of our sample was found to have reached this threshold (see Table 1).

A paired *t*-test was carried out to check whether the participant population's results for the Smartphone Addiction Scale tests had significantly changed between briefing (M=89.39,SD=23.41) and debriefing sessions (M=86.28,SD=19.22). This was not the case (p=.077) and the test values were highly correlated amongst themselves (r=.807,n=64,p<.001). For this reason, we used the mean of both tests for the analyses. The age and gender of participants are also taken into account in this study.

During the course of the study, the bespoke Tymer app functioned on each participant's device to accomplish two things. Firstly, to prompt the user for feedback on their current mood state (not featured in this analysis) and secondly, to track user interactions with their device. Each interaction is referred to as an *event*, recorded alongside a time stamp and the source of the event (i.e., the application with which the user interacts). The participants used Tymer for an average of 53.08 days (SD = 18.74).

**Fig. 1:**
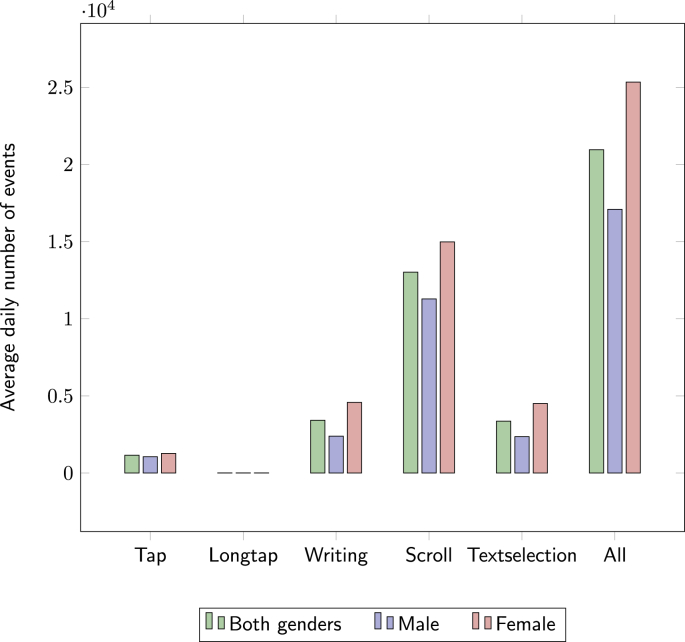
Average number of daily events per event type and gender.

Only UI interaction events were considered, excluding events that could be ambiguous (i.e., triggered by either the user or the smartphone, such as a screen off event, that can be caused by the user switching off the screen but also by the smartphone timing out the display). This was done to avoid including smartphone generated events as they do not constitute a real UI interaction, but also to exclude passive usage (e.g., streaming a video, using the phone as navigation system) which can count towards screen time, but does not constitute active interaction with the smartphone. The specific types of events considered are: tap, long tap, writing (e.g., striking the keyboard), scroll, and text selection (i.e., highlighting). We subsequently refer to the sum of all of these events together as “overall smartphone use”. Note large numbers of events were recorded over the period (Fig. 1), with scrolling making a particularly significant contribution.

**Fig. 2:**
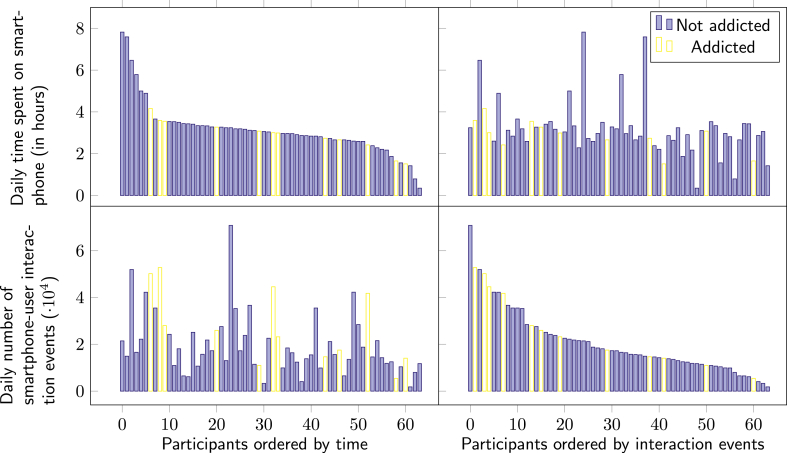
Daily usage of non-addicted and addicted users, N=64.

**Fig. 3:**
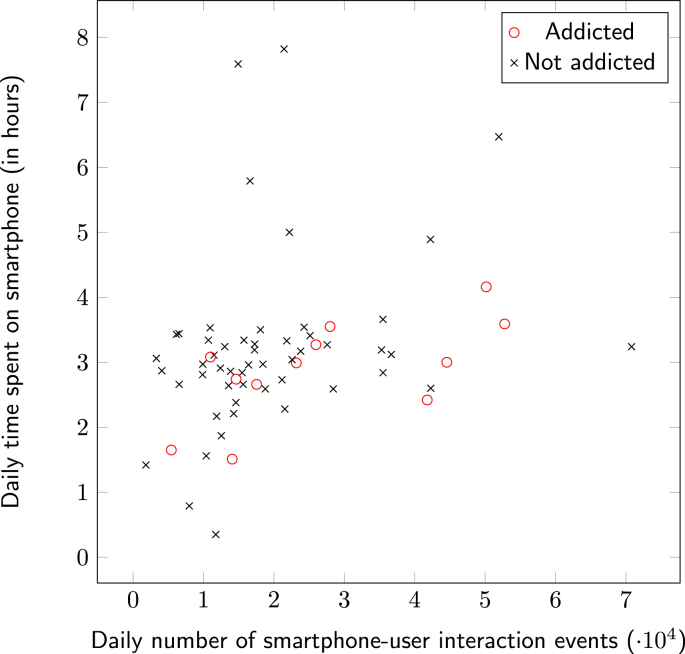
Daily usage of non-addicted and addicted users by time spent on phone, n=64.

In Fig. 2 we present descriptive statistics concerning smartphone usage, for users classified by their addiction status, as determined by the SAS-SV (Kwon et al., 2013a). In the left panel, users are ordered by descending daily time spent on the smartphone, and notably, addiction is widely spread across the distribution. The same data is presented in the right panel, but with the users ordered by decreasing number of daily UI interactions. Here there appears to be increased clustering of SA for users at the head of the distribution. The relationship between daily time on the smartphone and UI interactions is further expressed in Fig. 3. Note that for the smartphone addicted sub-group, there is much greater variance in UI interactions, as compared to daily time on the smartphone.

### 3.1 Analysis design

From comparing the number of events between participants, we analyse differences in participants' usage behaviour. The sum of events was chosen as the principle variable rather than a total session time length since it requires no interpretation of the data and represents the intensity of smartphone-user engagement. The data was normalised to obtain the average daily number of events, to account for any deviations in participation, such as from choosing to opt out or a delayed start. It should be noted that different apps generate different types and volumes of events. However this is not an issue in this study since we are principally concerned with analysing inter-personal differences relating to the same app or category of app.

**Fig. 4:**
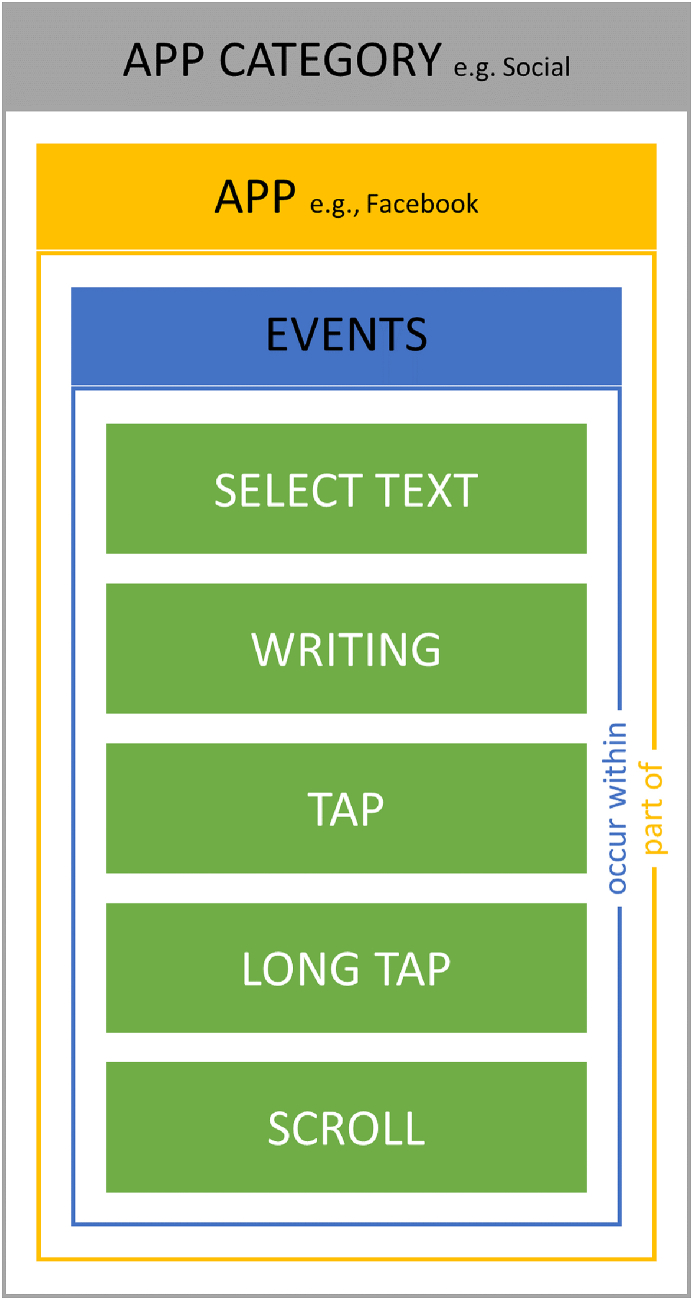
The relationship between events, apps and categories of smartphone applications.

Fig. 4 shows how user behaviour can be captured through total events of different types, that occur within different apps belonging to different categories of apps. These provide different levels of aggregation for a user's interaction and we consider their correlation with SA.

**Fig. 5:**
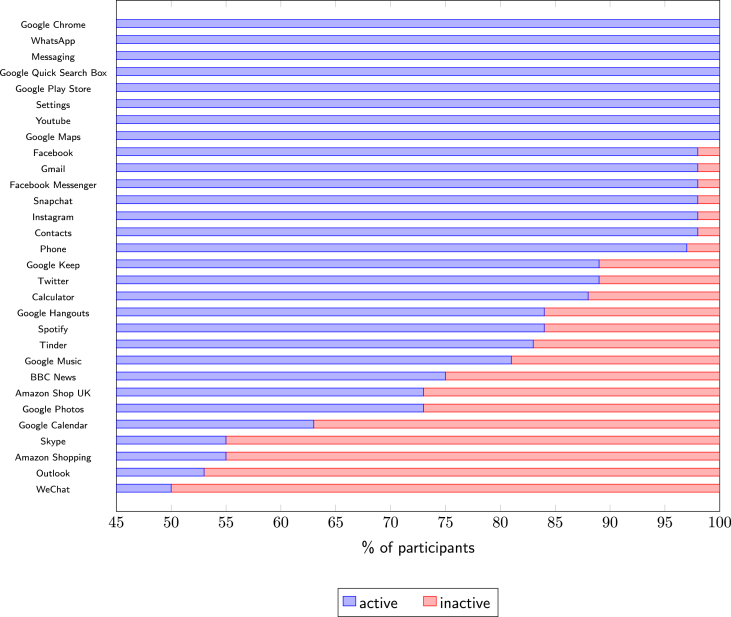
Percentage of participants having at least one recorded UI interaction event, i.e., being active on popular apps, n=64.

Events within app categories capture broad user behaviour, and can be modelled by the approximate taxonomy provided by the Google Play Store. No changes were made to these categories, with the exception of game app subcategories that were aggregated into a single Game category. Uncategorised apps were not included in the analyses pertaining to app categories. To allow cross comparison, app categories used by less than half of the participants were excluded, resulting in a list of 21 app categories. Considering events within apps provides insight into behaviour aligned with a particular bundle of functionality. Again, only sources from popular apps were included; apps that were absent from at least half of the participant's data and brand specific apps were excluded, resulting in the consideration of 29 apps (see Fig. 5). The total number of events across all apps was computed for each event type in isolation. These are potentially indicative of variations in overall usage between users. The types of events considered relate to a tap, long tap, writing, scroll, and text selection.

Gender and age are known characteristics likely to influence smartphone usage (De-Sola Gutiérrez et al., 2016; Roberts et al., 2014). To account for these confounding factors in each analysis, partial Spearman correlations with SA were first carried out separately for each gender, using age as a covariate. If the correlation coefficient was of the same sign, the analysis was repeated combining both male and female participants, and using both age and gender as covariates.

To correct for possible occurrence of type I errors, a False Discovery Rate (FDR) method was applied using the Benjamini-Hochberg procedure (Benjamini & Yosef, 1995).

## 4 Results

Participants received a mean score of 87.84 (SD=20.28) on the SAS. Neither gender was more or less likely to be addicted to smartphones than the other ($t\left(62\right)=.257,\;p=.614$). The mean SA score obtained by male participants was 89.01 (SD=21.10), while it was 86.51 (SD=19.57) for female participants. Moreover, age was not significantly correlated to SA (r=.219,n=64,p=.082).

When the daily amount of time spent on smartphones was evaluated in relation to SA, no significant association was found for males $\left(r=-.073,\;p=.685\right)$, females $\left(r=.351,\;p=.062\right)$, and across gender $\left(r=.150,\;p=.244\right)$. This formalises the observations made on the descriptive statistics in Section 3 (Fig. 2) where SA is seen across participants with wide ranging daily time on the smartphone. This may indicate that passive usage, characterised by high time periods using the smartphone with low levels of UI interaction, could be a common use case in smartphone usage. This provides evidence that alone, time on the smartphone does not necessarily provide a strong indicator of problematic smartphone behaviour, and further motivates the analysis of UI interaction as an indicator of SA, as considered in the following analyses.

### 4.1 UI interactions in app categories and Smartphone Addiction

From considering total events by app category, analysis was undertaken based on gender, assessing each gender in isolation and also together (Table 2). These analyses allow investigation of Hypotheses *H2* and *H3*.

**Table 2:** Application categories use correlation with Smartphone Addiction for male (a), female (b), and all users (c).

App Categories	M		F		M+F	
	r	p	r	p	r	p
Auto And Vehicles	.230	.198	.013	.948	.105	.416
Business	.066	.713	.228	.234	.159	.216
Communication	.100	.581	.011	.953	.048	.711
Entertainment	.068	.708	.075	.700	.068	.601
Finance	.068	.708	.187	.332	.200	.118
Game	.062	.730	.093	.631	.109	.401
Health And Fitness	.131	.468	-.066	.732		
Lifestyle	.230	.199	.680	.000^∗^^a^	.446	.000^∗^^a^
Maps And Navigation	.284	.109	.337	.074	.322	.011^∗^
Music And Audio	.007	.971	.169	.381	.062	.630
News And Magazines	.205	.252	.195	.310	.198	.122
Personalization	.111	.537	.270	.157	.151	.241
Photography	.165	.360	.011	.957	.117	.364
Productivity	.148	.412	.036	.851	.120	.353
Shopping	.064	.724	.142	.464	.085	.510
Social	.364	.037^∗^	.445	.016^∗^	.421	.001^∗^^a^
Sports	.234	.191	.014	.944	.123	.341
Tools	.495	.003^∗^	-.117	.546		
Travel And Local	.177	.323	.040	.835	.143	.268
Video Players	.098	.586	.085	.661	.090	.488

^∗^p < .05.

a FDR < .05.

For female participants, SA was linked to Lifestyle (r=.680,p<.001) apps. When both genders were considered together, correlations were found with Lifestyle (r=.446,p=.001) and Social (r=.421,p=.001) app categories. This is consistent with previous findings (Montag et al., 2015a) concerning social media addiction.

These results support Hypothesis *H2*, however a significant correlation with social apps only occurs when combined analysis is performed on gender; this is likely due to limitation in sample size for the gender specific analyses. Interestingly, the results do not support *H3*, with the Communication app category not being significantly correlated to SA. It may well be that the multifaceted functionality of social media is permitting new channels through which traditional text based-communication can be undertaken.

### 4.2 UI interactions within apps and Smartphone Addiction

While considering the total UI interaction events generated in each app in isolation, analysis was undertaken based on gender, assessing each gender separately, followed by analyses across genders. Table 3 shows the correlation scores for the apps relevant to Hypotheses *H2* and *H3* and from the app categories that were found to be significantly correlated to SA. Consistent with the previous analysis of app categories, none of the Communication apps were significantly correlated to SA. However, Snapchat, an app from the Social category, was found to be significantly correlated to SA (r=.386,p=.002). This is an interesting finding, consistent with Snapchat providing more than only functional user experience relating to pleasure and entertainment. However, in contrast to previous research (Punyanunt-Carter et al., 2017), addiction to Snapchat appears more significant than as for other social media applications.

**Table 3:** Application use correlation with Smartphone Addiction for male (a), female (b), and all users (c) for a subset of analysed apps.

Apps	M		F		M+F	
	r	p	r	p	r	p
**Communication**						
Contacts	.459	.007^∗^	-.182	.344		
Facebook Messenger	.204	.254	-.129	.505		
Gmail	-.037	.840	-.033	.867	-.037	.777
Google Chrome	.177	.325	-.135	.486		
Google Hangouts	.084	.642	.126	.516	.058	.655
Skype	.045	.805	-.318	.092		
WeChat	.094	.602	.281	.139	.183	.155
WhatsApp	.159	.376	.331	.079	.210	.102

**Social**						
Facebook	.324	.066	.176	.361	.309	.014^∗^
Instagram	.358	.041^∗^	-.017	.931		
Snapchat	.326	.064	.548	.002^∗^	.386	.002^∗^^a^

**Lifestyle**						
Tinder	.086	.634	.203	.292	.121	.348

^∗^p < .05.

a FDR < .05.

These results partially support Hypothesis *H2*, but the non-significant findings for the social media applications Facebook and Instagram were not foreseen. While Snapchat is not classified in the app store as a Communication application, aspects of its functionality lend themselves to a process of targeted information exchange, lending some partial but weak support for Hypothesis *H3*.

It is also notable that, while positive correlations were found for the Lifestyle category, Tinder, the only app analysed in isolation from this category had no significant correlation to SA.

### 4.3 UI event types and Smartphone Addiction

To investigate Hypothesis *H1*, UI interaction events were considered by each different type (tap, long tap, writing, scroll, text selection and all combined, see Table 4). When both genders were combined, overall usage (r=.280,p=.028) and scrolling (r=.284,p=.025) events were found to be positively correlated to SA. However, these results were not significant for FDR < 0.05. This partially supports Hypothesis *H1*.

**Table 4:** Correlation between app usage per event type and Smartphone Addiction for male (a), female (b), and all users (c) for a subset of analysed apps for which at least one p < .05 result was found.

Event type	App	M		F		M+F	
		r	p	r	p	r	p
All	all	.303	.087	.253	.186	.280	.028^∗^
Tap	all	.170	.344	.241	.207	.226	.078
	Contacts	.427	.013^∗^	-.187	.331		
	Facebook	.363	.038^∗^	.103	.596	.307	.015^∗^
	Google Quick Search Box	.344	.050^∗^	.242	.207	.315	.013^∗^
	Instagram	.345	.049^∗^	-.004	.984		
	Snapchat	.322	.068	.645	.000^∗^^a^	.459	.000^∗^^a^
Long tap	all	.142	.431	.241	.207	.210	.101
	Contacts	.485	.004^∗^^a^	-.069	.720		
	Facebook	.321	.068	.105	.587	.260	.042^∗^
	Google Quick Search Box	.284	.109	.307	.106	.283	.026^∗^
	Snapchat	.240	.178	.498	.006^∗^	.368	.003^∗^^a^
Writing	all	-.053	.770	.327	.083		
	Contacts	.496	.003^∗^	-.226	.239		
	Facebook	.273	.124	.198	.304	.259	.042^∗^
	Google Play Store	.320	.069	.240	.210	.277	.030^∗^
	Google Quick Search Box	.412	.017^∗^	-.067	.728		
	Snapchat	.281	.113	.620	.000^∗^^a^	.432	.000^∗^^a^
	Spotify	.106	.557	.481	.008^∗^	.261	.040^∗^
Scroll	all	.406	.019^∗^	.159	.410	.284	.025^∗^
	Facebook	.285	.024^∗^	.159	.409	.295	.020^∗^
	Google Quick Search Box	.265	.037^∗^	-.066	.734		
	Settings	.359	.004^∗^	.246	.198	.326	.010^∗^
	Snapchat	.346	.006^∗^	.459	.012^∗^	.347	.006^∗^
	WhatsApp	.260	.042^∗^	.322	.088	.276	.030^∗^
Text selection	all	.123	.497	.295	.120	.142	.270
	Contacts	.419	.015^∗^	-.343	.068		
	Facebook	.373	.032^∗^	.119	.537	.287	.024^∗^
	Google Play Store	.404	.020^∗^	.168	.385	.316	.012^∗^
	Google Quick Search Box	.456	.008^∗^	-.074	.705		
	Snapchat	.323	.067	.536	.003^∗^	.411	.001^∗^^a^
	Spotify	.153	.396	.418	.024^∗^	.262	.039^∗^

^∗^p < .05.

a FDR < .05.

We note that even though tap, long tap, writing, and text selection events were not significantly associated with SA when all app data was considered, they were in the context of Snapchat. This is likely due to the overall strong correlation of Snapchat usage, regardless of the interaction type. This is especially true for female users.

Interestingly, long tap events (used to bring up context menus) in the Contacts built-in application were significantly correlated to SA for male users only. The strong correlations found for other event types (albeit not significant for FDR < .05) suggest that this result might be due to the overall correlation of Contacts use and SA. No significant correlations were however found for scroll events, which is of particular interest as they are the most used type of UI interaction overall (see Fig. 1). We also note that no significant correlation could be established for female users.

These results further provide some support for Hypotheses *H1* and *H2*, with weak support for *H3* when male usage of the Contacts app and the communication function of Snapchat is considered. Again it is notable that as compared to Facebook and Instagram, interaction with Snapchat has a more statistically significant correlation.

## 5 Discussion

The results provide some evidence for Hypothesis *H1*, noting that overall usage and scrolling events appear positively correlated to SA, when taken on aggregate (Section 4.3), although they do not withstand FDR correction. When considering events by their source application (Section 4.2), Snapchat was repeatedly found to be an origin from which particular events significantly positively correlate with SA. This also supports social media being a source of high activity for smartphone addicts (Hypothesis *H2*). Indeed, we find that across the categories of apps (Section 4.1), level of engagement (i.e., total user-generated events) in the Social category significantly correlates with SA, in support of Hypothesis *H2*. It is notable that the Lifestyle category is also significantly correlated with SA (Section 4.1). However, the findings in support of *H2* are limited to correlation with activity in Snapchat, rather than a broader range of social media applications. This appears to provide a differential between Snapchat and other forms of social media, which was not anticipated based on the previous literature. Little support for Hypothesis *H3* was found, which may be due to social media apps implicitly providing communication functionalities. However, usage of popular messaging apps, such as WhatsApp, was not found to be significantly correlated with SA.

Correlation between Lifestyle apps and SA was not hypothesised to be significant. Tinder is the only Lifestyle app that was analysed in isolation in our app analyses, however no significant relationship was found between its use and SA, for both genders. Given the diversity of potential Lifestyle apps, it is therefore difficult to establish any common characteristics of the Lifestyle apps used that contribute to correlation with SA, especially in the case of female users (r=.680). Since Lifestyle apps are diverse and indeed linked to a certain lifestyle that might widely differ per individual, it is perhaps the higher interaction with personalised, non-mainstream apps that is the basis of SA in this context. The gender difference might be explained by the greater brand commitment women showcase (Tifferet & Ram, 2012), the difference between how male and female users personalise their phones (Tossell, Kortum, Shepard, Ahmad, & Zhong, 2012), and that a large portion of top apps seem to target topics which in general, women display more interest in than men (Daly, Hogg, Sacks, Smith, & Zimring, 1983; Fichten & Sunerton, 1983).

Focusing on the findings for social media however, the results suggest that assessment of the user's device interaction is a useful proxy for Smartphone Addiction assessment. Importantly, the results show utility in observing UI interactions at a detailed level, and provide a means to filter false positive results that occur due to passive use of the smartphone. Our findings are consistent with the general literature that correlates social media usage with SA (David et al., 2015; Enez Darcin et al., 2016; Jeong et al., 2016; Ryan et al., 2014; Salehan & Negahban, 2013) and the fear of missing out (FoMO) (Alt, 2015; Przybylski, Murayama, DeHaan, & Gladwell, 2013), which has been previously aligned with problematic usage (Elhai et al., 2016). However, the prominence of Snapchat, relative to other social media apps, is a particularly interesting aspect in our results. This is significant because Snapchat is heavily used in the wider population, and is attractive to teenagers (Statista, 2016). While wider concerns have been voiced in the wider press on the addictive nature of Snapchat (Andersson, 2018; Kallie, 2018; Stewart, 2018; White, 2017), to the best of our knowledge, limited academic research has been conducted to date (Punyanunt-Carter et al., 2017).

### 5.1 Pathways to addiction

Smartphone usage provides pathways to addiction on several distinct levels. Firstly, the ease of access combined with the widespread and socially accepted use of smartphones, makes smartphone use ubiquitous. Secondly, the increasing number of functionalities smartphone (apps) offer, make users more reliant on the technology and incentivise smartphone usage over analog options or other digital devices. Thirdly, apps are designed to make users prolong their usage (e.g., through “infinite scrolling”, which lacks any stopping cues) or come back to them (e.g., notifications or daily rewards urging the user to open up the app). In our fine-grained analysis of UI interaction events within applications, we have attempted to look more closely at this third phenomenon. Observing UI interaction directly engages the potential problematic behaviour as a signal. This also reduces the opportunity for false positive problematic use classification to arise for users who have high levels of passive usage, such as occurs through streaming media or using the smartphone as a GPS device.

Beyond interaction with the device, the bundled nature of apps gives a multitude of ways through which app design and user experience may further contribute to problematic behaviour. FoMO is applicable across all social media apps and therefore characteristics beyond this are relevant. In particular, it is conceivable that the particular design of the Snapchat app contributes to the significant correlations that are repeatedly evident in our analyses, as compared to other social media apps (e.g., Facebook, Instagram).

We note that Snapchat is distinct from other social media in providing multimedia messaging whose main feature is the ephemeral nature of the text, picture or video messages. Snapchat has a combination of design features that promote high frequency usage. For example, “friend emojis” appear next to a user's friends' names only if they “snap” each other regularly and feedback is provided on when content has been consumed by the recipient. The app's functionality, in terms of filters used to visually edit content, also regularly changes. The self-deleting nature of content and the way in which messages are sent creates a perception of control and privacy (Utz, Muscanell, & Khalid, 2015). Incentives are also provided for users to increase their snapping frequency (i.e., send new content), by effectively making friends compete against each other for top position and incentivising them not to break their “Snapstreak” (which is only maintained if both interaction partners have sent each other a Snap within the last 24 hours). This latter form of gamification promotes usage, but it may also promote addiction (Hellman, Schoenmakers, Nordstrom, & Van Holst, 2013). Additionally, in contrast to some other social media platforms, Snapchat is only available for smartphones (as opposed to a general web application), channelling all usage through the handset.

Interestingly, the Snapchat app also connects with entertainment aspects of social communication, with “chatting through pictures” providing a strong emotional context as compared to text (Vaterlaus, Barnett, Roche, & Young, 2016). The combined need for the app to support fun and functionality has been previously linked to Snapchat addiction (Punyanunt-Carter et al., 2017), when Snapchat usage is considered in isolation. Based on our findings, we hypothesise that the design features of Snapchat, relative to other social media apps, are providing a strengthened environment for SA to take hold.

### 5.2 Limitations

Although equal or greater in sample size than other observational and task based studies (e.g. (Andrist, Ruis, & Shaffer, 2018; Marquet, Alberico, & Hipp, 2018; Peleg-Adler, Lanir, & Korman, 2018; Wang & Hung, 2018)), it is possible that, given the power of the present study, we did not detect all true associations, and thus replications with larger samples may reveal additional findings. We also note that due to the properties of different smartphone operating systems, only Android users were selected for this study. The nature of data collection through smartphones, based on an app, means that data collection was beyond our immediate control, leading to exclusion of certain participants or missing data (e.g., due to a low battery preventing the app from recording and/or sending data). Lastly, we acknowledge that this study was conducted through a non-clinical sample, recruited largely in a higher education environment. We thus would not infer that the smartphone usage levels and/or SAS scores indicate any impairment of everyday functioning.

## 6 Conclusion

We have introduced a new approach to monitoring user activity for the detection of problematic smartphone behaviour, by considering all the UI interactions made by a user. As compared to other measures, this directly engages the potential problematic physical behaviour as a signal. This approach also allows for a focus on exclusively active rather than passive smartphone activity. Our data indicates that the time a smartphone is active may not be a significant correlate of problematic behaviour, which is symptomatic of the smartphone being used for an increasingly wide variety of applications. Within heavy and diverse usage, a more detailed analysis of behaviour allows the potential sources of addiction to be better understood. Scrolling behaviour in particular, is worthy of further investigation.

Through this approach, using an exploratory study, we have been able to analyse indicators of Smartphone Addiction across a sample of smartphone users (N=64) over a period of 8 weeks. This has identified that Smartphone Addiction is associated with the usage of Lifestyle and Social applications. We have also discovered that Snapchat, one of the most popular social media applications in use today, may be particularly indicative of problematic smartphone usage, indicated across all types of UI interactions. To date, few academic studies have identified such issues and this is worthy of further consideration, particularly given the significant usage of Snapchat by teenagers. Preliminary analysis of the Snapchat app, compared to other social media, leads us to hypothesise that the app's design provides a particularly strong pathway towards Smartphone Addiction.

Research on the addictive elements of these apps, and in particular of Snapchat, is worth pursuing. Determining the direction of causality and further examining social and environmental factors that could be at play would be of great interest.

## Conflicts of interest

The authors declare that they have no competing interests.
